# Sickle Cell Disease in Latin America and the Caribbean: Where the Care Cascade Breaks Down

**DOI:** 10.7759/cureus.116316

**Published:** 2026-09-15

**Authors:** María Betzabeth Orozco Rodríguez, Juan David Urrea Ospina, Daniel Felipe Facundo Solarte, Karen Jimena Oviedo Restrepo, Laura Sofía Montealegre Neiza, María Paula Salazar Cedeño, María Ángel Zarama Silva, Juan David Gomez Andrade, Juan Jose Martinez Peñaranda

**Affiliations:** 1 School of Medicine, Universidad Libre de Colombia, Cali, COL; 2 Department of Internal Medicine, Universidad Libre de Colombia, Interinstitutional Internal Medicine Group (GIMI 1), Cali, COL

**Keywords:** access to care, caribbean region, hydroxyurea, latin america, neonatal screening, sickle cell anemia, stroke, transcranial doppler ultrasonography

## Abstract

Sickle cell disease causes stroke in childhood and shortens life expectancy. Whether interventions that modify its course reach patients in Latin America and the Caribbean, and where the sequence fails when they do not, remains poorly characterized. This narrative review examines that question for neonatal screening, transcranial Doppler, and hydroxyurea, treating them as a care cascade with steps of detecting the disease, stratifying stroke risk, and reaching disease-modifying therapy. PubMed/MEDLINE, Scopus, Web of Science, and Scientific Electronic Library Online (SciELO) were searched for records published from 1998 to August 2026, and studies were retained when they reported regional data on at least one step of the cascade or on the burden of the disease and access to care in the region. The evidence identified indicates that the sequence breaks at every step, for different reasons at each. Neonatal screening appears widespread as a national program, although the panels do not always include this disease, and detection and linkage to care emerge as separate achievements. For transcranial Doppler, the reviewed evidence shows the widest gap between the strength of the efficacy data and the unevenness of implementation, both between countries and within them. Reported proportions of hydroxyurea use cannot be compared with one another because the denominators behind them differ, and beyond hydroxyurea, the regional evidence for newer therapies is close to absent. Beyond access, the same reports rarely state which ultrasound modality and which genotype-specific reference values were applied, even though both determine the risk category a given velocity falls into. On the evidence identified, closing these gaps appears to depend less on new technology than on population denominators that allow coverage to be expressed against eligible patients, and on an explicit statement of method in the studies the region publishes.

## Introduction and background

Sickle cell disease causes ischemic stroke in childhood and shortens life expectancy by decades, and stroke is the complication whose prevention is both established and measurable. Worldwide, the number living with the disease rose by 41.4% between 2000 and 2021, and Latin America and the Caribbean is one of only two superregions, with sub-Saharan Africa, where incidence at birth rose over that period; in Jamaica it ranks among the three leading causes of death in children under five [1]. Treatment meanwhile expanded beyond hydroxyurea with crizanlizumab [2] and gene therapy, whose estimated minimum cost exceeds 2.1 million U.S. dollars [3].

We treat the three interventions as a care cascade: each step conditions the next, so that detecting the disease, stratifying stroke risk, and reaching disease-modifying therapy must all succeed for any to change an outcome. None of the three is recent, and all were validated in populations and health systems unlike those of the region [4].

The scope is deliberately narrow: the disease affects nearly all systems, and covering them all would prevent us from following any one through to the patient. This review therefore asks one question: at which step of that sequence does the cascade break down in Latin America and the Caribbean, and what does the published evidence show about why. We did not identify a previous regional review that follows these three interventions as a single sequence, and the check behind that statement is reported with the search methods; that is what this review adds, because a cascade shows at which step the sequence breaks and by how much.

The population is reaching adulthood while neonatal screening and transcranial Doppler remain pediatric interventions, and the Brazilian protocol restricts the examination to patients aged two to 16 [5], although the median age at death in a Brazilian national series now falls in the fourth decade [6]. The cascade therefore ends where its evidence ends, at the transfer to adult care, a phase the regional literature barely describes [7].

## Review

Review question and objectives

Stated in full: for patients with sickle cell disease in Latin America and the Caribbean, how far have neonatal screening, transcranial Doppler, and hydroxyurea reached the patient, and where does the published evidence place the break in that sequence. Three objectives follow, one per step: to describe the availability and coverage of neonatal screening and the linkage of detected infants to care; to describe the coverage of transcranial Doppler and the reference values applied to classify its results; and to describe the reported use of hydroxyurea and the regional evidence for the therapies that came after it. Within each step, availability, coverage, adherence, and clinical effectiveness are reported separately where the evidence allows, because a country may run a program and still not reach its patients, and every proportion is given with the denominator behind it. Complications outside these three steps, and the management of acute events, are outside the scope.

Methods of the literature search

Search Strategy

We searched PubMed/MEDLINE, Scopus, Web of Science, and the Scientific Electronic Library Online (SciELO), combining controlled vocabulary terms with their free-text equivalents across three conceptual blocks, linked with “OR” within each block and with “AND” between blocks. Each Medical Subject Headings (MeSH) term was verified against the official vocabulary prior to use. For the disease, we used “Anemia,” “Sickle Cell,” and “Hemoglobin, Sickle”; for the region, “Latin America,” “South America,” “Central America,” “Caribbean Region,” and “Mexico”; and for the care continuum, descriptors such as neonatal and mass screening, late diagnosis, transcranial Doppler ultrasonography, stroke, blood flow velocity, neuropsychological testing, hydroxyurea, anti-sickle cell agents, blood transfusion, gene therapy, hematopoietic stem cell transplantation, and access to care were used.

The same concepts were searched for as free text in titles and abstracts using the “Title/Abstract” field in PubMed, “TITLE-ABS-KEY” in Scopus, “TS” in Web of Science, and their equivalents in Spanish and Portuguese in SciELO, including “anemia falciforme,” “doença falciforme,” “triagem neonatal,” and “doppler transcraniano.”

The main string combined the three blocks with AND, and two further strings complemented it. A string of the disease and region blocks alone, without the cascade block, recovered 1,013 records that the main string did not. A third string paired the disease block with the country of author affiliation, using the Affiliation field, and recovered a further 1,264 that neither of the other two retrieved. That third route is what surfaces regional studies that never describe themselves as Latin American in their title or abstract, and several of the regional studies cited here were retrieved only by it.

The lower cutoff was 1998, the year in which the Stroke Prevention Trial in Sickle Cell Anemia established transcranial Doppler velocity as the basis for stroke risk stratification [4]; this cutoff did not exclude any of the references cited here, since the oldest among them was that very trial. Studies in Spanish, English, and Portuguese were included, and the search covered records available from that date through August 19, 2026.

Screening, Full-Text Retrieval, and Sources

The searches identified 10,793 records: 3,965 from PubMed/MEDLINE across the three strings, 3,335 from Scopus, 3,108 from Web of Science, and 385 from SciELO. Removing duplicate records within and between databases left 4,814, of which two were set aside because their complete bibliographic record could not be retrieved. The remaining 4,812 were screened by title and abstract against four criteria: the record concerns sickle cell disease; it was published from 1998 onward; it reports a regional signal, whether through indexing terms, title or abstract, or author affiliation; and it addresses at least one of the three steps of the cascade or, failing that, the burden of the disease or access to care in the region. Letters, editorials, comments, and errata were excluded. That screening was distributed among three pairs of authors, six in total, with every record read independently by both members of its pair and discrepancies resolved by consensus. Screening retained 1,618 records, of which 854 address a step of the cascade and 764 the burden of the disease or access to care alone.

Full text was sought for 1,409 of those records and obtained for 716. Of the 693 that were sought and not obtained, 528 had no open copy reported by the publisher and 165 had one that could not be retrieved. Retrieval was not attempted for the remaining 209, of which 181 came from database exports that carried neither a digital object identifier nor a PubMed identifier. Records whose full text was obtained were not assessed against a second set of eligibility criteria; the sources cited are those that report regional data on a step of the cascade or on the burden that frames it, selected for their relevance to the argument, as the Limitations state. Every retrieved article used as a source was matched to its record by the identifier printed on its first page and by its title before any figure was taken from it.

This review cites 33 references. Twenty-eight of them are records retained at screening, and five were identified outside the search, which was built to find regional studies rather than the documents that frame them: the 1998 trial that fixes the lower limit of the search window, a worldwide review of newborn screening programs, and three programmatic or regulatory documents, a national screening report and two national instruments, a resolution and a law. Those three are cited to establish that a program exists and what its panel contains, never as evidence of effect. To document the absence stated in the introduction, we also examined the 76 retained records whose titles identify them as reviews: none of them follows the three interventions as a single sequence, and each of those with a regional scope concerns a single country or territory.

Design, Quality Appraisal, and Data Extraction

This is a narrative review and is reported as such, and it was not conducted as a systematic review. No protocol was registered in advance, although the search strategy was written out in full before the searches were run; no formal risk-of-bias instrument was applied to the individual studies; and no quantitative synthesis or meta-analysis was planned or performed. Nothing here is reported as compliance with Preferred Reporting Items for Systematic Reviews and Meta-Analyses (PRISMA), and no flow diagram is presented, because the counts above give the same figures that a diagram would display. The decision not to pool follows from the heterogeneity of the evidence identified, whose populations, denominators, study designs, and moments of measurement differ to a degree that makes pooling uninterpretable, as the section on hydroxyurea sets out.

Study quality was appraised qualitatively, and the appraisal governed how much weight a finding carried rather than whether it entered the review. Four things were recorded for each source: whether it is an original study or a synthesis of other studies; whether it is a national registry or a multicenter series rather than a single-center report; whether it states the denominator behind each proportion; and whether the figure quoted is the one the study reports or one calculated by a later review. National registries and multicenter series that state their denominators carry more interpretive weight here than single-center reports that do not, and where a figure was calculated by a previous systematic review rather than reported by the original study, the text says so where it appears. Data were extracted by the same three pairs of authors, independently within each pair, with discrepancies resolved by consensus; each claim was recorded against the persistent identifier of its source, and each quantitative statement was checked against the full text rather than against the abstract alone, except for one series of adults whose full text could not be retrieved and whose figures were checked against its published abstract, which reports all of them. Every effect estimate is reproduced as its source reports it, with the confidence interval and the P value where the source gives them, and every proportion is reported with the denominator behind it, or with a note where its source does not state one. Table 1 reproduces the complete search strategy for each database.

**Table 1:** Complete search strategy by database Field tags for PubMed/MEDLINE are shown in parentheses, for example (MeSH) and (tiab), because this journal reserves square brackets for citations; the database itself requires square brackets in those positions. The asterisk is the truncation operator. The three PubMed searches were run by the authors through E-utilities; those in Scopus, Web of Science, and Scientific Electronic Library Online (SciELO) were run in each portal and their results exported. In Web of Science the year range was applied through the results panel rather than inside the string. Uppercase words that are not search terms are the Boolean operators AND and OR, or database field tags: TITLE-ABS-KEY, AFFILCOUNTRY, and PUBYEAR in Scopus; TS and CU in Web of Science; and MeSH, tiab, and Affiliation in PubMed/MEDLINE. Among the search terms, TCD denotes transcranial Doppler and Hb SS denotes homozygous sickle cell disease. Source: Prepared by the authors.

Database	Search	Query
PubMed/MEDLINE	Main	((("Anemia, Sickle Cell"(MeSH) OR "Hemoglobin, Sickle"(MeSH)) OR ("sickle cell"(tiab) OR "sickle-cell"(tiab) OR sicklemia(tiab) OR drepanocytosis(tiab) OR drepanocytic(tiab) OR "drepanocitose"(tiab) OR "anemia falciforme"(tiab) OR "doenca falciforme"(tiab) OR "anemia drepanocitica"(tiab) OR HbSS(tiab) OR HbSC(tiab) OR "hemoglobin S"(tiab) OR "haemoglobin S"(tiab) OR "Hb SS"(tiab)))) AND ((("Latin America"(MeSH) OR "South America"(MeSH) OR "Central America"(MeSH) OR "Caribbean Region"(MeSH) OR "Mexico"(MeSH)) OR ("Latin America"(tiab) OR "Latin American"(tiab) OR "Latin-America"(tiab) OR Caribbean(tiab) OR "South America"(tiab) OR "South American"(tiab) OR "Central America"(tiab) OR "Central American"(tiab) OR Iberoamerica*(tiab) OR Brazil*(tiab) OR Brasil*(tiab) OR Colombia*(tiab) OR Mexic*(tiab) OR Argentin*(tiab) OR Peru(tiab) OR Peruvian(tiab) OR Chile*(tiab) OR Venezuela*(tiab) OR Ecuador*(tiab) OR Bolivia*(tiab) OR Paraguay*(tiab) OR Uruguay*(tiab) OR Cuba(tiab) OR Cuban(tiab) OR Jamaica*(tiab) OR Haiti*(tiab) OR "Dominican Republic"(tiab) OR Panama*(tiab) OR "Costa Rica"(tiab) OR Guatemala*(tiab) OR Hondura*(tiab) OR Nicaragua*(tiab) OR "El Salvador"(tiab) OR Guyana(tiab) OR Suriname(tiab) OR "Trinidad and Tobago"(tiab) OR Belize(tiab) OR "Puerto Rico"(tiab) OR "French Guiana"(tiab)))) AND ((("Neonatal Screening"(MeSH) OR "Mass Screening"(MeSH) OR "Early Diagnosis"(MeSH) OR "Delayed Diagnosis"(MeSH) OR "newborn screening"(tiab) OR "neonatal screening"(tiab) OR "heel prick"(tiab) OR "early detection"(tiab) OR "late diagnosis"(tiab) OR "delayed diagnosis"(tiab) OR "triagem neonatal"(tiab) OR "tamizaje neonatal"(tiab) OR "cribado neonatal"(tiab)) OR ("Ultrasonography, Doppler, Transcranial"(MeSH) OR "Stroke"(MeSH) OR "Blood Flow Velocity"(MeSH) OR "Neuropsychological Tests"(MeSH) OR "transcranial Doppler"(tiab) OR "transcranial doppler"(tiab) OR TCD(tiab) OR stroke(tiab) OR "cerebrovascular"(tiab) OR "silent infarct"(tiab) OR "cerebral infarct"(tiab) OR "neurocognitive"(tiab) OR "cognitive"(tiab) OR "doppler transcraniano"(tiab) OR "doppler transcraneal"(tiab)) OR ("Hydroxyurea"(MeSH) OR "Genetic Therapy"(MeSH) OR "Blood Transfusion"(MeSH) OR "Antisickling Agents"(MeSH) OR "Hematopoietic Stem Cell Transplantation"(MeSH) OR hydroxyurea(tiab) OR hydroxycarbamide(tiab) OR hidroxiureia(tiab) OR hidroxiurea(tiab) OR voxelotor(tiab) OR crizanlizumab(tiab) OR "L-glutamine"(tiab) OR "gene therapy"(tiab) OR "gene editing"(tiab) OR exagamglogene(tiab) OR lovotibeglogene(tiab) OR "stem cell transplant"(tiab) OR "chronic transfusion"(tiab) OR "exchange transfusion"(tiab)) OR ("Health Services Accessibility"(MeSH) OR "Healthcare Disparities"(MeSH) OR "Health Equity"(MeSH) OR "Health Policy"(MeSH) OR "access to care"(tiab) OR "health care access"(tiab) OR "healthcare access"(tiab) OR "disparities"(tiab) OR "inequities"(tiab) OR "inequalities"(tiab) OR "coverage"(tiab) OR "health policy"(tiab) OR "acesso"(tiab) OR "acceso"(tiab))))
PubMed/MEDLINE	Complementary, disease and region only	((("Anemia, Sickle Cell"(MeSH) OR "Hemoglobin, Sickle"(MeSH)) OR ("sickle cell"(tiab) OR "sickle-cell"(tiab) OR sicklemia(tiab) OR drepanocytosis(tiab) OR drepanocytic(tiab) OR "drepanocitose"(tiab) OR "anemia falciforme"(tiab) OR "doenca falciforme"(tiab) OR "anemia drepanocitica"(tiab) OR HbSS(tiab) OR HbSC(tiab) OR "hemoglobin S"(tiab) OR "haemoglobin S"(tiab) OR "Hb SS"(tiab)))) AND ((("Latin America"(MeSH) OR "South America"(MeSH) OR "Central America"(MeSH) OR "Caribbean Region"(MeSH) OR "Mexico"(MeSH)) OR ("Latin America"(tiab) OR "Latin American"(tiab) OR "Latin-America"(tiab) OR Caribbean(tiab) OR "South America"(tiab) OR "South American"(tiab) OR "Central America"(tiab) OR "Central American"(tiab) OR Iberoamerica*(tiab) OR Brazil*(tiab) OR Brasil*(tiab) OR Colombia*(tiab) OR Mexic*(tiab) OR Argentin*(tiab) OR Peru(tiab) OR Peruvian(tiab) OR Chile*(tiab) OR Venezuela*(tiab) OR Ecuador*(tiab) OR Bolivia*(tiab) OR Paraguay*(tiab) OR Uruguay*(tiab) OR Cuba(tiab) OR Cuban(tiab) OR Jamaica*(tiab) OR Haiti*(tiab) OR "Dominican Republic"(tiab) OR Panama*(tiab) OR "Costa Rica"(tiab) OR Guatemala*(tiab) OR Hondura*(tiab) OR Nicaragua*(tiab) OR "El Salvador"(tiab) OR Guyana(tiab) OR Suriname(tiab) OR "Trinidad and Tobago"(tiab) OR Belize(tiab) OR "Puerto Rico"(tiab) OR "French Guiana"(tiab))))
PubMed/MEDLINE	Author affiliation	((("Anemia, Sickle Cell"(MeSH) OR "Hemoglobin, Sickle"(MeSH)) OR ("sickle cell"(tiab) OR "sickle-cell"(tiab) OR sicklemia(tiab) OR drepanocytosis(tiab) OR drepanocytic(tiab) OR "drepanocitose"(tiab) OR "anemia falciforme"(tiab) OR "doenca falciforme"(tiab) OR "anemia drepanocitica"(tiab) OR HbSS(tiab) OR HbSC(tiab) OR "hemoglobin S"(tiab) OR "haemoglobin S"(tiab) OR "Hb SS"(tiab)))) AND ("Brazil"(Affiliation) OR "Brasil"(Affiliation) OR "Colombia"(Affiliation) OR "Mexico"(Affiliation) OR "México"(Affiliation) OR "Argentina"(Affiliation) OR "Peru"(Affiliation) OR "Perú"(Affiliation) OR "Chile"(Affiliation) OR "Venezuela"(Affiliation) OR "Ecuador"(Affiliation) OR "Bolivia"(Affiliation) OR "Paraguay"(Affiliation) OR "Uruguay"(Affiliation) OR "Cuba"(Affiliation) OR "Jamaica"(Affiliation) OR "Haiti"(Affiliation) OR "Dominican Republic"(Affiliation) OR "Panama"(Affiliation) OR "Panamá"(Affiliation) OR "Costa Rica"(Affiliation) OR "Guatemala"(Affiliation) OR "Honduras"(Affiliation) OR "Nicaragua"(Affiliation) OR "El Salvador"(Affiliation) OR "Guyana"(Affiliation) OR "Suriname"(Affiliation) OR "Trinidad and Tobago"(Affiliation) OR "Belize"(Affiliation) OR "Puerto Rico"(Affiliation) OR "French Guiana"(Affiliation))
Scopus	Main	TITLE-ABS-KEY("sickle cell" OR "sickle-cell" OR sicklemia OR drepanocyt* OR "drepanocitose" OR "anemia falciforme" OR "doenca falciforme" OR "anemia drepanocitica" OR HbSS OR HbSC OR "hemoglobin S" OR "haemoglobin S") AND TITLE-ABS-KEY("Latin America*" OR Caribbean OR "South America*" OR "Central America*" OR Iberoameric* OR Brazil* OR Brasil* OR Colombia* OR Mexic* OR Argentin* OR Peru OR Peruvian OR Chile* OR Venezuela* OR Ecuador* OR Bolivia* OR Paraguay* OR Uruguay* OR Cuba OR Cuban OR Jamaica* OR Haiti* OR "Dominican Republic" OR Panama* OR "Costa Rica" OR Guatemala* OR Hondura* OR Nicaragua* OR "El Salvador" OR Guyana OR Suriname OR "Trinidad and Tobago" OR Belize OR "Puerto Rico" OR "French Guiana") AND TITLE-ABS-KEY("newborn screening" OR "neonatal screening" OR "triagem neonatal" OR "tamizaje neonatal" OR "cribado neonatal" OR "heel prick" OR "early detection" OR "late diagnos*" OR "delayed diagnos*" OR "transcranial Doppler" OR "doppler transcrania*" OR "doppler transcrane*" OR TCD OR stroke OR cerebrovascular OR "silent infarct*" OR neurocognit* OR cognitive OR hydroxyurea OR hydroxycarbamide OR hidroxiure* OR voxelotor OR crizanlizumab OR "L-glutamine" OR "gene therapy" OR "gene editing" OR exagamglogene OR lovotibeglogene OR "stem cell transplant*" OR "chronic transfusion" OR "exchange transfusion" OR "access to care" OR "healthcare access" OR disparit* OR inequit* OR inequalit* OR coverage OR "health polic*") AND PUBYEAR > 1997
Scopus	Author affiliation	TITLE-ABS-KEY("sickle cell" OR "sickle-cell" OR sicklemia OR drepanocyt* OR "anemia falciforme" OR "doenca falciforme" OR "anemia drepanocitica" OR HbSS OR HbSC OR "hemoglobin S" OR "haemoglobin S") AND AFFILCOUNTRY(Brazil OR Colombia OR Mexico OR Argentina OR Peru OR Chile OR Venezuela OR Ecuador OR Bolivia OR Paraguay OR Uruguay OR Cuba OR Jamaica OR Haiti OR "Dominican Republic" OR Panama OR "Costa Rica" OR Guatemala OR Honduras OR Nicaragua OR "El Salvador" OR Guyana OR Suriname OR "Trinidad and Tobago" OR Belize OR "Puerto Rico" OR "French Guiana") AND PUBYEAR > 1997
Web of Science	Main	TS=("sickle cell" OR "sickle-cell" OR sicklemia OR drepanocyt* OR "drepanocitose" OR "anemia falciforme" OR "doenca falciforme" OR "anemia drepanocitica" OR HbSS OR HbSC OR "hemoglobin S" OR "haemoglobin S") AND TS=("Latin America*" OR Caribbean OR "South America*" OR "Central America*" OR Iberoameric* OR Brazil* OR Brasil* OR Colombia* OR Mexic* OR Argentin* OR Peru OR Peruvian OR Chile* OR Venezuela* OR Ecuador* OR Bolivia* OR Paraguay* OR Uruguay* OR Cuba OR Cuban OR Jamaica* OR Haiti* OR "Dominican Republic" OR Panama* OR "Costa Rica" OR Guatemala* OR Hondura* OR Nicaragua* OR "El Salvador" OR Guyana OR Suriname OR "Trinidad and Tobago" OR Belize OR "Puerto Rico" OR "French Guiana") AND TS=("newborn screening" OR "neonatal screening" OR "triagem neonatal" OR "tamizaje neonatal" OR "cribado neonatal" OR "heel prick" OR "early detection" OR "late diagnos*" OR "delayed diagnos*" OR "transcranial Doppler" OR "doppler transcrania*" OR "doppler transcrane*" OR TCD OR stroke OR cerebrovascular OR "silent infarct*" OR neurocognit* OR cognitive OR hydroxyurea OR hydroxycarbamide OR hidroxiure* OR voxelotor OR crizanlizumab OR "L-glutamine" OR "gene therapy" OR "gene editing" OR exagamglogene OR lovotibeglogene OR "stem cell transplant*" OR "chronic transfusion" OR "exchange transfusion" OR "access to care" OR "healthcare access" OR disparit* OR inequit* OR inequalit* OR coverage OR "health polic*")
Web of Science	Author affiliation	TS=("sickle cell" OR "sickle-cell" OR sicklemia OR drepanocyt* OR "anemia falciforme" OR "doenca falciforme" OR "anemia drepanocitica" OR HbSS OR HbSC OR "hemoglobin S" OR "haemoglobin S") AND CU=(Brazil OR Colombia OR Mexico OR Argentina OR Peru OR Chile OR Venezuela OR Ecuador OR Bolivia OR Paraguay OR Uruguay OR Cuba OR Jamaica OR Haiti OR "Dominican Republic" OR Panama OR "Costa Rica" OR Guatemala OR Honduras OR Nicaragua OR "El Salvador" OR Guyana OR Suriname OR "Trinidad and Tobago" OR Belize OR "Puerto Rico" OR "French Guiana")
SciELO	Detection	("anemia falciforme" OR "doença falciforme" OR "doenca falciforme" OR "anemia drepanocítica" OR "sickle cell") AND ("triagem neonatal" OR "tamizaje neonatal" OR "cribado neonatal" OR "newborn screening" OR "teste do pezinho")
SciELO	Stroke risk stratification	("anemia falciforme" OR "doença falciforme" OR "anemia drepanocítica" OR "sickle cell") AND ("doppler transcraniano" OR "doppler transcraneal" OR "transcranial doppler" OR "acidente vascular cerebral" OR "accidente cerebrovascular" OR stroke)
SciELO	Disease-modifying therapy	("anemia falciforme" OR "doença falciforme" OR "anemia drepanocítica" OR "sickle cell") AND (hidroxiureia OR hidroxiurea OR hydroxyurea OR "terapia gênica" OR "terapia génica" OR "transplante de células-tronco" OR "transfusão crônica")
SciELO	Burden, access, and equity	("anemia falciforme" OR "doença falciforme" OR "anemia drepanocítica" OR "sickle cell") AND (mortalidade OR mortalidad OR prevalência OR prevalencia OR acesso OR acceso OR iniquidade OR inequidad OR "política de saúde" OR "política de salud")

The burden and who bears it

Recorded Mortality

In the two countries in the region for which we identified national data series, recorded mortality showed an increasing trend. In Colombia, the age-standardized mortality rate increased from 0.055 to 0.143 per 100,000 inhabitants between 2012 and 2023, based on 675 deaths recorded in the national death certificates. The rate ratio was 1.03 per year (95% confidence interval, CI 1.01-1.06; p = 0.002), with a particularly marked increase among men, whose average annual percentage change was 8.20% (95% CI 0.98-15.24; p = 0.029) [8]. In Brazil, a similar trend was observed over a longer period, with an average annual percent change of 4.58 (95% CI 3.73-5.43) based on 9,808 deaths between 1996 and 2019 [9].

However, both studies caution against a direct interpretation of this trend. The Brazilian study attributes it in part to improvements in the quality of the mortality reporting system, while the Colombian study notes that the observed increase cannot be interpreted solely as evidence of rising mortality [8,9]. Two decades after the launch of national neonatal screening, the evidence we identified does not distinguish a real increase in deaths from an improvement in record-keeping.

Colombian data show where recorded mortality is concentrated. The highest average annual mortality rates are concentrated in the Caribbean departments, led by Bolívar at 0.386 per 100,000 inhabitants, La Guajira at 0.341, and Magdalena at 0.334, compared to 0.029 in Bogotá; among the 10 departments with the highest rates, the ratio between the extremes was 13.3 to 1 [8]. Departments with low socioeconomic development had three times the mortality rate of those with high development (rate ratio, RR 3.00; 95% CI 1.19-7.61; p = 0.020), and four times higher after adjusting for sex and area of residence (RR 4.00; 95% CI 1.60-9.97; p = 0.003) [8]. The authors emphasize that this gradient cannot be interpreted as a causal effect independent of poverty, because the general population denominators do not allow for distinguishing between a higher concentration of affected individuals, poorer survival, reduced access to care, or differences in registration [8].

At the individual level, the Brazilian data quantify the gap. The median age at death among people with sickle cell disease was 32.0 years, compared with 69.0 years in the general population, a difference of 37 years, and between the first and the ninth year of life the risk of death was more than 30 times that of the general population (RR 32.78) [6].

What the Records Miss

Two findings suggest that these figures are conservative. In a Brazilian state program that screened 3.6 million newborns, the term sickle cell was not recorded on 46% of the 193 death certificates of children with a known diagnosis [10]. Globally, cause-specific deaths in 2021 totaled 34,400 (95% uncertainty interval, UI 25,000-45,200), while the total mortality burden reached 376,000 (95% UI 303,000-467,000), which the same analysis describes as nearly 11 times higher [1]. That analysis also reported that Latin America and the Caribbean, along with sub-Saharan Africa, is one of only two superregions where incident cases at birth are increasing, by 5.6% (95% UI 2.1-10.5) [1].

Figure 1 presents the care cascade that organizes the remainder of this review, showing the regional figure associated with each step and the country of origin.

**Figure 1:**
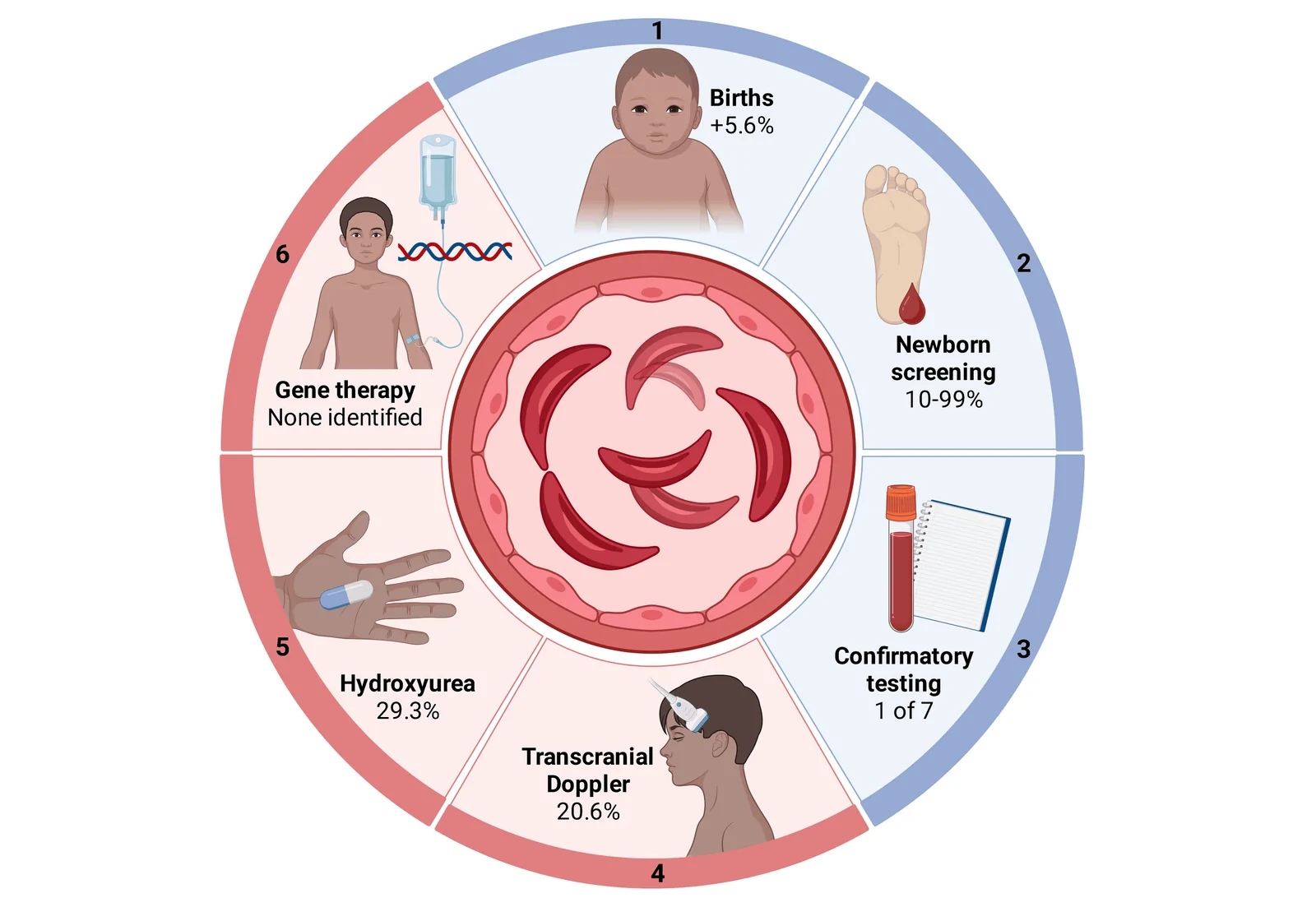
The care cascade for sickle cell disease in Latin America and the Caribbean The sectors follow the clinical order clockwise starting from the top left, and each sector is labeled with the regional figure we identified. (1) Births, with a 5.6% increase in incidence between 2000 and 2021 [1]. (2) Neonatal screening, ranging from over 99% in Martinique according to 2019 data [11] to 10.2% in Colombia in 2024 [12]. (3) Diagnostic confirmation, with one in seven positive results confirmed in Guyana [13]; the link to care also failed in a Brazilian state [14] and at a rural hospital in Haiti [15]. (4) Transcranial Doppler, 20.6% national coverage in Brazil [16]. (5) Hydroxyurea, administered to 29.3% of children and 36.3% of adults in a Brazilian multicenter cohort [17]. (6) Gene therapy, with no evidence of regional use among the studies we identified. The percentages come from different countries, years, and denominators, and are not directly comparable with one another. Source: Prepared by the authors based on the cited references. The figure was assembled manually using the BioRender icon library by Urrea Ospina, J. D. (2026).

Detecting the disease: neonatal screening

Clinical Effectiveness

Screening works, but direct evidence supporting its effectiveness is scarcer than the practice itself. A systematic review identified a single study with direct evidence, a retrospective historical-control study conducted in Jamaica, and rated by the review itself as having a high risk of bias, in which mortality in the first five years of life was 0.02% among screened children against 0.19% among those not screened; the odds ratio, calculated by the review itself rather than reported by the original study, was 0.09 (95% CI 0.04-0.22; p < 0.001), and at 10 years the difference was no longer significant (0.33; 95% CI 0.07-1.64; p = 0.176) [18]. In contrast, a Brazilian program covering 3.6 million newborns found no reduction in mortality between children born in the first seven and those born in the last seven years, with seven-year death rates of 5.43% and 5.12%, respectively (p = 0.72). The authors concluded that screening, however comprehensive it may have been, was insufficient [10].

Linkage to Care

Both results are consistent once the intermediate step is examined: detection and treatment are distinct achievements, and regional literature measures the gap between them. In Guyana, which did not have a screening program, a feasibility study found seven positive results for sickle cell anemia among 2,039 screened newborns and reported that only one was ultimately confirmed because the families lived in rural areas and the contact information proved unreliable [13]. In one Brazilian state, 27 of 302 detected cases (8.9%) were not being treated at the referral center, while another 20 (6.6%) were being treated but did not appear in the state laboratory’s database, meaning that the registry failed in both respects [14]. At a rural hospital in Haiti, five out of nine newborns with positive screening results returned for follow-up when point-of-care testing was available, compared with none out of six when it was not available (p = 0.044). At three of the program’s hospitals, the return rate ranged from 33% to 62% [15].

Coverage

Coverage differed widely between programs. A 2019 regional review reported coverage exceeding 98% in Guadeloupe and Jamaica and 99% in Martinique, against 79% and 45% in the pilot programs of Grenada and Saint Lucia; the review gives these as program coverage without stating the denominator behind each [11]. A worldwide review of newborn screening programs reports that Jamaica reached population-wide coverage in 2015 without a legislative mandate, and that the pilot programs in Grenada and in Saint Vincent and the Grenadines were discontinued for lack of funding and staff [19]. The contrast matters more than the range itself, because it separates programs with a standing budget from programs whose coverage ended with their funding.

Availability and Panel Content

In continental America, the question is not whether a national program exists, but what it aims to achieve. According to the same worldwide review, Panama has screened for hemoglobinopathies by law since 2007 as part of a mandatory national panel, and Mexico and Venezuela have had national screening regulations since 1988 and 1999 respectively, without hemoglobinopathies on either panel [19]. Colombia included them in its 2019 screening law, but they only became a mandatory and funded service through a ministerial resolution in January 2026 [20]; in 2024, the national screening report recorded 45,204 samples screened for hemoglobin disorders against 441,612 live births registered in the country, which is 10.2% [12]. The Dominican Republic follows the same pattern, with a five-year lag: its 2024 law incorporates hemoglobin disorders into mandatory basic screening, yet we could not locate the regulations that the law itself mandated be issued within ninety days [21].

Cuba stands apart because its national program does not screen newborns but prevents birth. The same regional review reports that screening for hemoglobinopathies has been conducted in pregnant women since 1982 and mandated by law since 1983, and that by December 2016, 7,659 at-risk couples had been identified, of whom 76.5% requested termination of pregnancy [11]. This represents a fourth logic, distinct from the cascade described in this review.

This brings us full circle to the data on disease burden: Colombia can measure its mortality but not its prevalence, which is precisely why its national study had to use denominators from the general population and why its geographical gradient does not allow for a causal interpretation [8,12].

Table 2 summarizes the content of the screening panel for each country and what the identified evidence establishes for the other two steps [2,5,11-17,19-25].

**Table 2:** Status of implementation of the three steps of the care cascade, by country or territory In the neonatal screening column, the existence and content of the panel are derived from the national standard or an international registry, not from our literature search. In the other two columns, NR indicates that our search did not find any studies, not that the activity is absent. The figures in different rows come from different years and denominators and are not comparable with one another. Source: Prepared by the authors based on references [2,5,11-17,19-25].

Country or territory	Neonatal screening	Transcranial Doppler	Modifying therapy
Brazil	Yes; nationwide in phases since 2001	20.6%; six states without public facilities	Hydroxyurea in 29.3% of children and 36.3% of adults
Colombia	10.2% by 2024; mandatory starting in January 2026	NR	62% without treatment in one community
Cuba	Not neonatal; national prenatal program since 1982	Six series identified	NR
Grenada	Pilot study not continued	NR	NR
Guadeloupe	Yes; universal since 1984, coverage exceeding 98%	NR	NR
French Guiana	Yes; part of the French national program	NR	NR
Guyana	No; feasibility study only	NR	NR
Haiti	Hospital-based; point-of-care testing	NR	NR
Jamaica	Yes; entire population since 2015, without a legal mandate	Identified cohorts; site of a trial	Seven patients in the pivotal trial
Martinique	Yes; coverage exceeding 99%	NR	NR
Mexico	Not on the panel; national standard since 1988	NR	NR
Panama	Yes; mandatory on the panel since 2007	NR	NR
Dominican Republic	Required by law since 2024; no regulations identified	Prospective program; 283 children	Hydroxyurea at the maximum tolerated dose
Saint Lucia	Yes; since 1992	NR	NR
Venezuela	Not on the panel; national standard since 1999	NR	NR

Stratifying stroke risk: transcranial Doppler

Coverage

Of the three interventions we reviewed, transcranial Doppler has the most well-established evidence of efficacy and the most uneven regional implementation. In Brazil, the country that accounts for 843 of the 1,618 retained records, coverage between 2018 and 2021 was 20.6% nationwide and 20.8% in rural areas of the Northeast; the study reproduces both figures from ministry sources and does not state the denominator behind them [16]. National outpatient records illustrate the extent of this shortfall: between January and August 2019, the public system recorded 15,090 people aged 0-19 with the disease and 3,072 tests, and in the North region, only one test was performed for 1,410 registered patients, while six states had no equipment available in the public health system between 2018 and 2020 [5].

Individual studies measure the same gap from the patient’s perspective. Among 42 children in northeastern Brazil, 18 (42.9%) had never undergone screening before the study, and 13 of those 18 were older than six years [16]. In a series of 396 children from nine centers, 114 (28.8%) underwent their first screening during the study at a mean age of 9.19 years, 101 of those 114 (88.6%) had already exceeded the recommended age, and five of them (4.4%) had already suffered a stroke [26]. In a birth cohort of 461 children with SC hemoglobinopathy, only 71 (15.4%) were eventually screened, and one girl suffered an ischemic stroke at age 11 without having been evaluated prior to the event [27].

Effect on Treatment Decisions

When screening was performed, it led to changes in treatment. In the Dominican Republic, a prospective program screened 283 children, and Figure 2 shows the distribution of their velocity categories; six months of hydroxyurea at a fixed dose of 20 mg/kg per day reduced blood flow velocities by an average of 20 cm/s and normalized those of 34 of the 56 hydroxyurea-naive children who had a six-month examination (61%), and titration to the maximum tolerated dose decreased sickle cell-related events (incidence rate ratio 0.59; 95% CI 0.36-0.98; p = 0.042) [22]. In the only randomized trial we identified with sites in Latin America and the Caribbean, conducted in Rio de Janeiro, Kingston, and Memphis among 1,234 screened children, the intention-to-treat analysis found no significant differences in the cumulative incidence of conversion from conditional to abnormal velocity at 15 months (9% vs. 47%; p = 0.16), based on 22 randomized patients out of the 100 required by the power calculation; the mean velocity decreased with hydroxyurea and increased without it (-15.5 vs. +10.2 cm/s; p = 0.02) [28].

**Figure 2:**
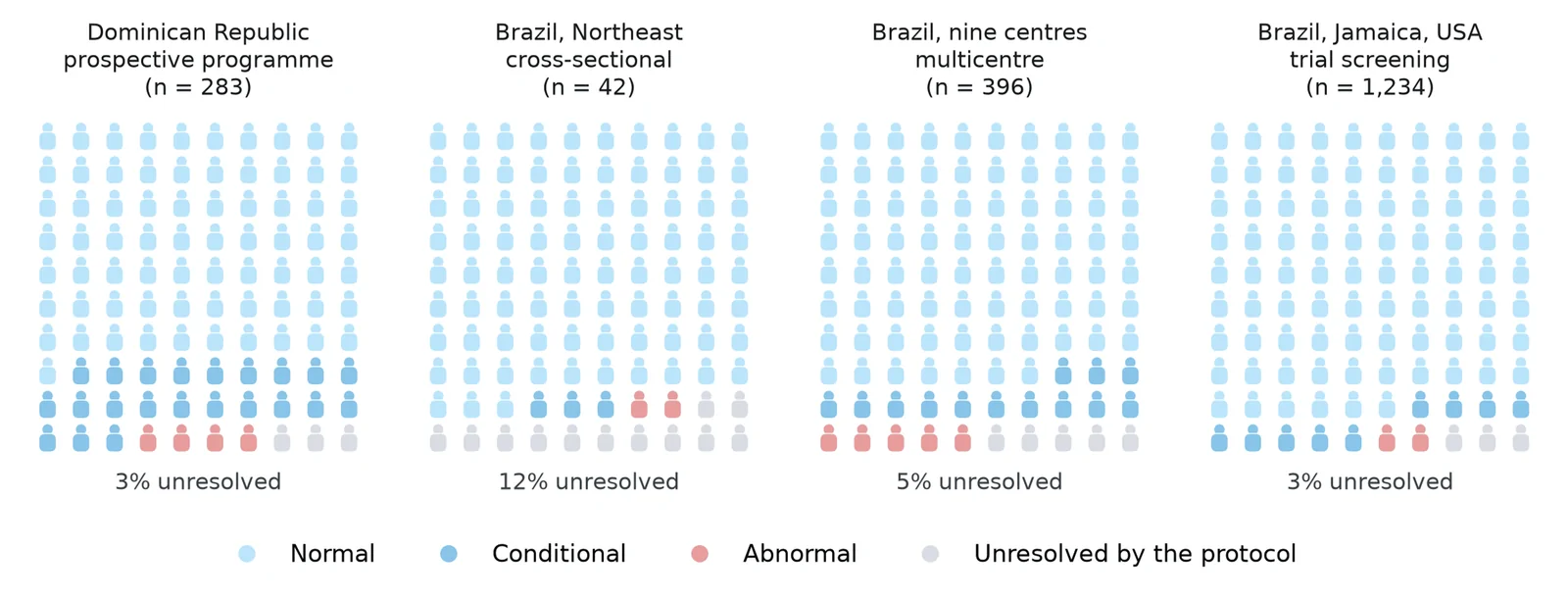
Distribution of velocity categories by transcranial Doppler in regional series reporting all categories Each icon represents 1% of examinations. The four identified series that report all categories instead of collapsing them are shown. The categories the original Stroke Prevention Trial in Sickle Cell Anemia protocol does not resolve, at the end of each matrix, comprise low velocity, inconclusive or inadequate studies, and technical failures. They correspond to a prospective program in the Dominican Republic (n = 283) [22], a cross-sectional series from northeastern Brazil (n = 42) [16], a Brazilian nine-center series (n = 396) [26], and the screening phase of a randomized trial in Brazil, Jamaica, and the United States (n = 1,234) [28]. The figure was created using Matplotlib (Python). Source: Prepared by the authors based on the cited references.

This trial also documents the difficulty of implementing it and partly explains why it was terminated early. An 18-month regulatory delay prevented the study drug from being imported to the Brazilian site, and two of the 11 children assigned to hydroxyurea never actually received it [28].

Which Countries Are Represented

The regional map does not follow a simple income gradient. We identified six Cuban primary studies from a single institute in Havana, published between 2009 and 2017 and indexed without a digital object identifier and without retrievable full text; they are counted among the records we identified and are not cited here as evidence, so part of the Cuban contribution remains invisible in the databases routinely consulted. Several countries well represented in the general corpus contributed no transcranial Doppler study that we identified, Mexico foremost among them, with 31 retained records and none on the examination. That is an absence of published and indexed evidence, not evidence that the examination is not performed; the two cannot be told apart from the literature alone, and they are kept apart throughout this review.

The series differ in how they classify their examinations. Figure 2 compares the distribution of velocity categories across the four identified series that report them in full, and the categories the original protocol does not resolve, added together within each series, account for between 3% and 12% of the examinations.

Transcranial Doppler: which threshold applies and to whom

Modality and Genotype

The threshold defining an abnormal velocity is not a single number. Non-imaging and imaging-based transcranial Doppler require different cutoffs, because angle correction and other technical differences shift the equivalent value downward [4,29], so the same child can be classified as normal or abnormal depending on the equipment used. None of the regional studies we identified stated which modality was used, although several named the equipment and allowed it to be inferred; a series from northeastern Brazil described imaging equipment and applied the cutoff derived without imaging [16]. Figure 3 sets out the values involved.

**Figure 3:**
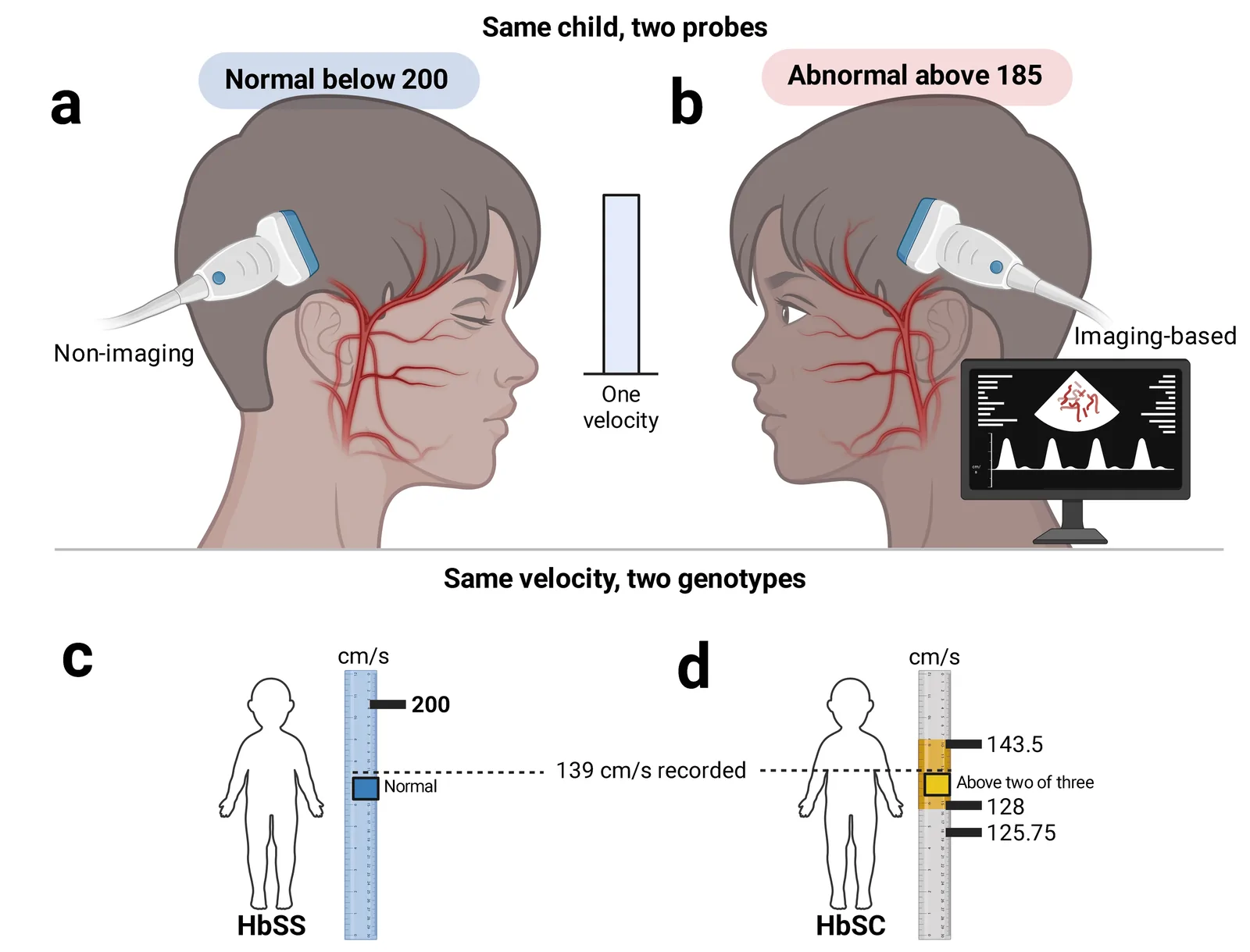
How the same measured velocity changes risk categories depending on the ultrasound modality and genotype The same velocity is classified differently depending on two decisions that regional studies rarely state. (a) Non-imaging transcranial Doppler uses the 200 cm/s criterion established in the original prevention trial [4]. (b) Imaging-based transcranial Doppler requires cutoff points 10% to 15% lower because of technical differences, particularly angle correction, and many centers adopt 185 cm/s [29], so that the same velocity measured in the same child changes category depending on the equipment used. (c) In homozygous disease, the reference value is that of the original trial and a velocity of 139 cm/s is classified as normal. (d) For SC hemoglobinopathy we did not identify established reference values, so those derived from homozygous disease are applied by default, even though thresholds of 128, 143.5, and 125.75 cm/s have been proposed [30]: that same velocity exceeds two of the three. Among the 71 children examined from a cohort of 461 with SC hemoglobinopathy, all classified as normal under the values for homozygous disease, the highest recorded velocity was 139 cm/s [27]. HbSS denotes homozygous sickle cell disease and HbSC denotes hemoglobin S combined with hemoglobin C. Source: Prepared by the authors based on the cited references. The figure was assembled manually using the BioRender icon library by Urrea Ospina, J. D. (2026).

The problem compounds in genotypes other than the homozygous one. We did not identify established reference values for SC hemoglobinopathy, so those derived from sickle cell anemia are applied by default even though lower thresholds have been proposed for it [30]. The consequence is visible in the cohort described above, where the children with SC hemoglobinopathy were classified as normal under homozygous values although their highest recorded velocity exceeded two of the three thresholds proposed for their genotype [27,30]. The same discrepancy reappears with age: among 50 adults followed at a Brazilian hematology outpatient clinic, the velocities associated with intracranial stenosis were lower than those described for children, and a threshold of 123.5 cm/s identified stenosis with 100% sensitivity and 73% specificity, and an area under the receiver operating characteristic curve of 0.91 (CI 0.79-1.00) [31].

Categories the Protocol Does Not Resolve

The categories the original protocol handles poorly are reported inconsistently and, among the studies we identified, are almost never analyzed. Across the regional series, which range from 42 to 1,234 examinations, they account for 1.0% to 9.5% of examinations as low velocity and for 2.4% to 4.3% as inconclusive, inadequate, or technically failed [16,22,26,28], and only four of the studies we identified separated them from the normal category. In a cohort from Minas Gerais, 54 children were classified as high risk, and nine of them were identified only by magnetic resonance angiography performed after an inadequate or inconclusive Doppler examination [32]. Those nine were found because the examination was followed up rather than recorded as normal.

Neurocognitive assessment runs into the same problem. A systematic review of low- and middle-income countries included 21 studies, seven of them from the region, four in Brazil and the rest in Cuba, Haiti, and the West Indies, and estimated a mean IQ of 83.8 (95% CI 79.9-87.7) in sickle cell disease, below normative expectations (z = -8.18; p < 0.0001), concluding that neurocognitive measures are routinely used outside the linguistic and cultural parameters for which they were developed [7].

Figure 3 shows how the same measured velocity shifts between risk categories depending on the ultrasound modality and the genotype-specific reference values applied to it; this is the mechanism common to both issues.

Modifying therapy: from hydroxyurea to gene therapy

Reported Use

Hydroxyurea is a therapy that has been studied in the region, and its reported use varies widely across studies, such that a single value cannot be established. In the Brazilian multicenter cohort of 2,795 participants, treatment was reported in 29.3% of the children and 36.3% of the adults at the time of enrollment [17]. At a single center in Rio de Janeiro in 2018, 614 of the 3,032 patients with at least one healthcare contact that year (20.3%) had hydroxyurea prescribed [33]. In a series of 42 children from the Northeast, 33 (78.5%) were taking it regularly [16]. In an Afro-Colombian community, 62% of the homozygous patients, eight in total, were not receiving it at the start of the study [25]. These proportions are not directly comparable: the denominators are a national cohort, all patients with a healthcare contact, a series from a specialized service, and eight homozygous individuals from a convenience sample.

Adherence

Where adherence is measured, it is measured by medication possession ratios: at that same center, 98 of the 614 patients with a prescription (16%) had a ratio above 50% [33], an indicator that does not distinguish between a patient who stops taking a medication and a system that fails to supply it. The evidence identified says less about the causes than those figures might suggest. The update to the Brazilian national guidelines expanded the criteria for hydroxyurea use, incorporated the 100-mg dosage into the public health system, and revised the text to avoid barriers to access; however, it states that no formal assessment of the facilitators and barriers to implementation was planned [23].

Newer Agents and Regulatory Status

Beyond hydroxyurea, this gap widens sharply. The region contributed substantially to the pivotal trial of crizanlizumab, with 40 of 198 patients recruited in Brazil and seven in Jamaica [2], and 90 of the 112 patients analyzed in a subsequent global managed-access program were Brazilian; they obtained the drug through compassionate use of a medication without local authorization rather than through the registration process [24]. This pathway was subsequently closed: the Brazilian regulatory agency revoked the marketing authorization on October 30, 2023, and the national health technology assessment of the drug was suspended without deliberation [23]. For voxelotor, we found no evidence of use, registration, or regional pricing in any country, and for L-glutamine, only isolated reports without denominators were found.

Curative Options

Curative therapies follow the same pattern. The Brazilian national guidelines recommend myeloablative allogeneic transplantation from an identical or haploidentical sibling donor in patients with SS or S-beta zero genotype and severe vaso-occlusive complications despite hydroxyurea, with human leukocyte antigen testing beginning at age five or upon the onset of the first symptoms [23]. An international Phase II trial of non-myeloablative haploidentical transplantation estimated its cost, based on published estimates, at approximately 432,157 U.S. dollars, compared to a minimum of 2.1 million for myeloablative gene therapy and gene editing [3]. Among the studies we identified, none reported gene therapy procedures performed in Latin America or the Caribbean.

Table 3 compares each disease-modifying option with the regional evidence identified for it.

**Table 3:** Disease-modifying options and regional evidence identified for each The costs are published estimates for other contexts and not regional prices. NR indicates that our search did not find the information, not that the option lacks regional evidence, regulatory registration, or a published price. Source: Prepared by the authors based on references [2,3,17,22-25,33].

Option	Mechanism	Regional evidence identified	Regulatory status	Estimated Cost
Hydroxyurea	Induction of fetal hemoglobin	20.3% to 78.5% depending on the series	Available, with national guidelines in Brazil	NR
Crizanlizumab	P-selectin inhibition	40 patients in Brazil, 7 in Jamaica	Registration canceled in Brazil, October 2023	NR
Voxelotor	Inhibition of hemoglobin polymerization	NR	NR	NR
L-glutamine	Reduction of oxidative stress	Isolated reports without common denominators	NR	NR
Allogeneic transplant	Curative, donor hematopoiesis	NR	Recommended in the Brazilian guidelines	$432,157 for the haploidentical modality
Gene therapy and gene editing	Curative, autologous modification	NR	NR	At least $2.1 million

Adult patients and the transition gap

The cascade described so far pertains to pediatric patients. Neonatal screening identifies infants, transcranial Doppler stratifies children, and the Brazilian protocol governing its use restricts the test to patients aged two to 16 years [5]. What happens to the patients saved by these interventions is the part of the cascade that the regional literature does not describe: among the studies we identified, none documented the transition from pediatric to adult care in Latin America or the Caribbean, and the review that mentions the problem supports its findings with cohorts from high-income countries, which are outside its own scope [7].

This omission matters because the burden shifts to adulthood three decades after these interventions take effect [6]: the patient saved by screening and transcranial Doppler reaches a stage of the system for which we could not identify a care model in the region.

Table 4 sets out each gap against the evidence that motivates it and the measure that would close it.

**Table 4:** Identified gaps and measures to close them SC refers to hemoglobin S combined with hemoglobin C. Source: Prepared by the authors based on references [5,7,8,11,16,27,29,30,32].

Gap	Identified evidence	Action that would address it
Denominators	Measured mortality compared to the general population	Prevalence estimates by country
Coverage metric	Reported cases relative to those tested, not to those eligible	Reported vs. eligible population
Ultrasound modality	Not reported in the identified studies	Mandatory reporting in each report
Thresholds by genotype	SC assessed using homozygous values	Regional validation by genotype
Unresolved categories	3% to 12% of tests, added together within each series	Separate report and clinical follow-up
Transition to adult care	No regional studies identified	Outcome cohorts following transfer

Limitations

This review describes the evidence identified through our search strategy and does not represent all existing evidence. Literature not indexed in the databases consulted, published in local journals, or unpublished material were excluded by design, and no conclusions presented here are intended to imply an absolute lack of evidence or to claim novelty.

Five limitations are specific. The search strategy retrieved indexed literature rather than regulatory instruments: the absence of studies from a given country tells us nothing about whether its programs exist. The cascade is a presentational tool rather than a measured pathway within a single population: the figures for each step come from different countries, study designs, and denominators, and should not be linked as if they described the trajectory of a single cohort. The literature we identified was heavily skewed toward Brazil, which accounted for 843 of the 1,618 retained records; therefore, regional claims rely disproportionately on a single country. Full-text articles were selected based on the relevance of their arguments rather than at random, which may have favored studies reporting quantified findings over those reporting none. Furthermore, some of the records retrieved from Scopus and SciELO lacked abstracts and were screened solely by title, a less sensitive procedure; the six Cuban studies on transcranial Doppler fall into a related category, as they are indexed without persistent identifiers and without a retrievable full text.

Where sources contained internal inconsistencies between their abstracts and tables, we used the values reported in the body of the article.

These gaps share a shape. Most close not with new technology or new studies but with declaration: stating the method that produced each figure, and expressing coverage against the population that should have been examined rather than against the patients who were. That common ground is what any comparison between countries in the region would require first.

## Conclusions

This review finds that the care cascade breaks down at all three steps examined, with differing causes for each. The common issue is not the absence of technology, but rather the lack of two prerequisites for improvement: population denominators that allow coverage to be expressed against eligible patients, and an explicit statement of the method by which each result was measured. This absence has consequences that extend beyond any individual study. Without a stated method, regional findings are difficult to compare and cannot contribute to revising the risk criteria applied by the region through transfer from other populations. The generation of therapies now arriving will be evaluated against standards defined outside the region, and this asymmetry will persist as long as the necessary information for discussion remains unreported in a systematic way.
